# Ribociclib for the first-line treatment of advanced hormone receptor-positive breast cancer: a review of subgroup analyses from the MONALEESA-2 trial

**DOI:** 10.1186/s13058-018-1050-7

**Published:** 2018-10-19

**Authors:** Gabriel N. Hortobagyi

**Affiliations:** Department of Breast Medical Oncology, Division of Cancer Medicine, The University of Texas MD Anderson Cancer Center, 1515 Holcombe Boulevard, Houston, TX 77030 USA

**Keywords:** CDK4/6 inhibitor, Ribociclib, Endocrine therapy, Hormone receptor-positive, HR^+^/HER2^−^ breast cancer, MONALEESA-2

## Abstract

**Abstract:**

Endocrine therapy is recommended for patients with hormone receptor-positive (HR^+^) advanced and metastatic breast cancer without visceral crisis (symptomatic visceral disease). However, many patients experience disease progression during treatment, and most patients eventually develop endocrine resistance. Therefore, it is important to identify treatment options that prolong the effectiveness of first-line endocrine therapies. Ribociclib is an orally bioavailable cyclin-dependent kinase (CDK) 4/6 inhibitor that has been approved for use in combination with an aromatase inhibitor for the treatment of HR^+^, human epidermal growth factor receptor 2-negative (HER2^−^) advanced breast cancer. This approval is based on findings from the MONALEESA-2 study, a double-blind, placebo-controlled, randomized phase 3 trial (NCT01958021) in which first-line therapy with ribociclib + letrozole significantly improved progression-free survival (PFS) compared with placebo + letrozole in patients with HR^+^/HER2^−^ advanced breast cancer. This review will discuss the overall findings from the MONALEESA-2 study and will provide a summarized analysis of results from the available subgroups in the study by age, visceral metastases, bone-only disease, de novo disease, and prior therapy. On the basis of these data, ribociclib has established itself as a beneficial treatment option for these different populations.

**Trial registration:**

ClinicalTrials.gov, NCT01958021. Registered on 8 October 2013.

## Background

Breast cancer accounts for 30% (252,710) of the new cancer cases and 14% (40,610) of cancer deaths in women in the US, according to statistics from 2017 [1]. Except in cases of visceral crisis, the standard of care in advanced hormone receptor-positive (HR^+^) breast cancer includes endocrine therapy (ET) alone or in combination with a targeted therapy [2]. However, many patients with newly diagnosed advanced breast cancer progress within a year of treatment with single-agent aromatase inhibitors [3], and the majority eventually develop endocrine resistance [4]. Therefore, identifying optimal first-line treatment options that delay disease progression in patients with locally advanced or metastatic cancer is critical [4].

### Available first-line options

The classification of trials as “first-line” is often unclear, as both patients with previous exposure to (neo)adjuvant ET and patients naive to ET are considered as receiving first-line treatment for advanced breast cancer [5]. However, factors such as differences in tumor biology, treatment approaches, and initiation and duration of prior treatment (if any) are significant considerations that inform treatment decisions [5]. To delay endocrine resistance, several therapies targeting the cyclin D1/cyclin-dependent kinase (CDK) pathway have been developed, including mammalian target of rapamycin (mTOR) inhibitors, phosphatidylinositol-3-kinase (PI3K) inhibitors/protein kinase B (AKT) inhibitors, and CDK inhibitors [6]. Targeting the CDK4/6 pathway through treatment with CDK4/6 inhibitors in combination with letrozole has led to significant improvement in progression-free survival (PFS) compared with that achieved with single-agent ET in first-line HR^+^ breast cancer [7, 8].

Currently, three CDK4/6 inhibitors, palbociclib, ribociclib, and abemaciclib, are approved by the US Food and Drug Administration (FDA) for use as first-line combination therapy with an aromatase inhibitor in the treatment of HR^+^/human epidermal growth factor receptor 2-negative (HER2^−^) advanced or metastatic breast cancer. These CDK4/6 inhibitors have been shown to significantly improve median PFS compared with endocrine monotherapy and/or placebo in randomized trials [7–9]. Ribociclib is an orally bioavailable small molecule that selectively inhibits CDK4/6, thereby inhibiting the phosphorylation of retinoblastoma protein, which prevents cell-cycle progression and arrests the cell cycle in the G_1_ phase [8]. In 2017 [10], ribociclib was approved by the US FDA on the basis of results from the phase 3 MONALEESA-2 trial of 668 patients with advanced breast cancer (ClinicalTrials.gov number, NCT01958021), in which treatment with ribociclib + letrozole met the PFS endpoint (hazard ratio (HR) = 0.56; 95% confidence interval (CI) 0.43–0.72) [8]. Initial US FDA approval of palbociclib was based on results from the phase 2 PALOMA-1 trial (NCT00721409), in which treatment with palbociclib + letrozole doubled the PFS compared with single-agent letrozole (20.2 vs 10.2 months, HR = 0.488, 95% CI 0.319–0.748, *P =* 0.0004) [11]. In the randomized (2:1 ratio) phase 3 PALOMA-2 study (NCT01740427), PFS in patients treated with palbociclib + letrozole was 24.8 months (95% CI 22.1 to not estimable) compared with 14.5 months (95% CI 12.9–17.1) in the placebo + letrozole group (HR = 0.58; 95% CI 0.46–0.72; *P* < 0.001), and the clinical benefit rate (CBR) was 84.9% vs 70.3% in the two groups, respectively [7]. First-line approval of abemaciclib + aromatase inhibitor was based on the randomized phase 3 MONARCH 3 trial (NCT02246621) in which abemaciclib + aromatase inhibitor significantly prolonged PFS vs placebo + aromatase inhibitor (median PFS: not reached vs 14.7 months; HR = 0.54; 95% CI 0.41–0.72; *P* = 0.000021) [9]. The CBR was 78.0% in the abemaciclib group vs 71.5% in the placebo group. Another available first-line treatment option for HR^+^ advanced breast cancer includes full-dose fulvestrant, a selective estrogen-receptor degrader [12]. In the randomized, double-blind phase 3 FALCON trial in patients with HR^+^ breast cancer, PFS was significantly prolonged with first-line fulvestrant 500 mg treatment (*n* = 230) compared with anastrozole (*n* = 232) (HR = 0.797; 95% CI 0.637–0.999; *P* = 0.0486), with a median PFS of 16.6 months vs 13.8 months, respectively [12].

### Overall results from MONALEESA-2

**Fig. 1:**
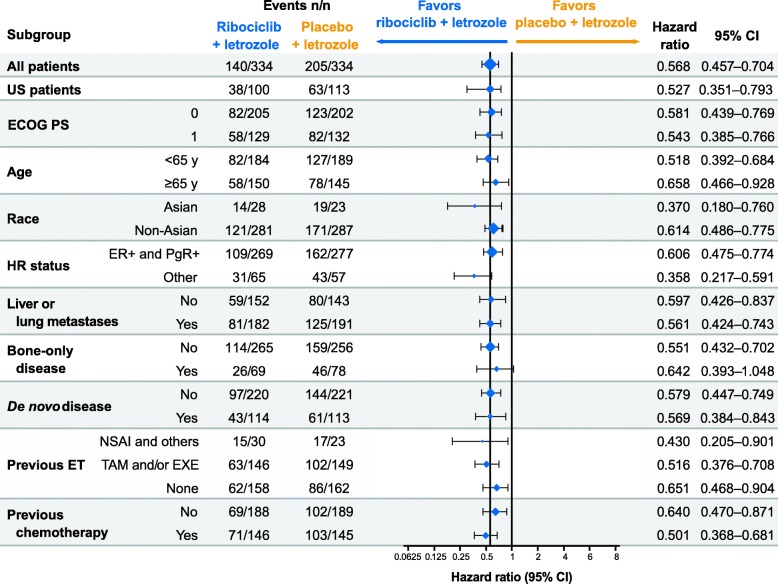
MONALEESA-2 subgroup analysis of locally assessed PFS. Data cut-off, 2 January 2017 (Hortobagyi GN et al. Updated results from MONALEESA-2, a Phase III trial of first-line ribociclib + letrozole in hormone receptor-positive, HER2-negative advanced breast cancer. Poster presented at the American Society of Clinical Oncology Annual Meeting, Chicago, IL, USA; 2–6 June 2017). CI confidence interval, ECOG PS Eastern Cooperative Oncology Group performance status, ER estrogen receptor, ET endocrine therapy, EXE exemestane, HR hormone receptor, NSAI nonsteroidal aromatase inhibitor, PFS progression-free survival, PgR progesterone receptor, TAM tamoxifen

MONALEESA-2 was an international, randomized, double-blind, placebo-controlled, phase 3 trial that has been described in detail previously [8]. The MONALEESA-2 trial was conducted in accordance with the Good Clinical Practice guidelines and the provisions of the Declaration of Helsinki. A total of 668 patients, from whom written informed consent had been obtained, were randomly assigned 1:1 to orally receive either ribociclib + letrozole or placebo + letrozole and were stratified by disease site (presence or absence of liver and/or lung metastases) [8]. Postmenopausal women with locally advanced or metastatic HR^+^/HER2^−^ breast cancer with ≥ 1 measurable lesion (Response Evaluation Criteria in Solid Tumors (RECIST) v1.1) or ≥ 1 predominantly lytic bone lesion and an Eastern Cooperative Oncology Group (ECOG) status of ≤ 1 were included [8]. Patients with any prior systemic therapy for advanced breast cancer (including ET or chemotherapy), inflammatory breast cancer, or active cardiac disease or history of cardiac dysfunction (corrected QT interval with Fridericia’s formula (QTcF) > 450 ms) were excluded [8]. At the initial interim analysis (data cut-off date, 29 January 2016), the trial met the primary endpoint of PFS. Patients in the ribociclib treatment group had a 44% lower relative risk of progression (*P* = 3.29 × 10^−6^) vs those in the placebo group. In the ribociclib treatment group, 195 patients (58%) remained on treatment vs 154 patients (46%) in the placebo group. Median PFS occurred at 14.7 months in the placebo group but was not reached in the ribociclib group due to continued treatment. The CBRs were 79.6% in the ribociclib group and 72.8% in the placebo group in the intention-to-treat population and 80.1% and 71.8%, respectively, in patients with measurable disease (*P* = 0.02 for both populations). The most common adverse events (AEs) occurring in ≥ 20% of the study population were neutropenia, nausea, infections, fatigue, diarrhea, alopecia, leukopenia, vomiting, arthralgia, constipation, headache, and hot flushes. The most common grade 3/4 AEs (> 3%) were neutropenia, leukopenia, abnormal liver function tests, infections, and vomiting. AEs leading to dose reductions of ribociclib occurred in 50.6% of patients receiving ribociclib + letrozole compared with 4.2% in patients receiving placebo + letrozole, and permanent discontinuation of ribociclib + letrozole due to AEs occurred in 7.5% of patients. The AE most frequently leading to dose reduction was neutropenia (*n* = 104/169 patients with dose reduction due to AE in the ribociclib group vs no patients in the placebo group). On-treatment deaths, regardless of causality, were reported in three patients (0.9%) treated with ribociclib + letrozole vs one patient (0.3%) treated with placebo + letrozole. Causes of death in patients taking ribociclib + letrozole were progressive disease, death (cause unknown), and sudden death (in the setting of grade 3 hypokalemia and grade 2 QT prolongation). The demographics of the populations included in the subgroup analyses of MONALEESA-2 were well-balanced, and median duration of the study in all subsets was ≥ 12 months. Median PFS and CBR results favored the ribociclib group across all predefined subgroups. The safety profile of ribociclib + letrozole was similar across all subsets (Table 1). Results from a second overall survival interim analysis (data cut-off, 2 January 2017) of MONALEESA-2 showed that the PFS benefit was maintained for ribociclib at 25.3 months vs 16.0 months for the placebo group (HR = 0.568; 95% CI 0.457–0.704; *P* = 9.63 × 10^−8^), with a consistent PFS benefit across patient subgroups (Hortobagyi GN et al. Updated results from MONALEESA-2, a Phase III trial of first-line ribociclib + letrozole in hormone receptor-positive, HER2-negative advanced breast cancer. Poster presented at the American Society of Clinical Oncology Annual Meeting, Chicago, IL, USA; 2–6 June 2017) (Fig. 1). However, this review will discuss results from the interim PFS data cut-off (29 January 2016) unless otherwise indicated.

**Table 1:** MONALEESA-2 safety profile: all-grade adverse events across studies (≥ 30% of patients in any group)

AE, n (%)	Age < 65 years (*n* = 370) [17]		Age ≥ 65 years^a^ (*n* = 294) [17]		Visceral metastases (*n* = 393) (Burris et al., 2016)		Bone-only disease (*n* = 146) (Burris et al., 2016)		*De novo* disease (*n* = 226) [21]		Prior CT (*n* = 289) (Conte et al., 2017)		No prior CT (*n* = 375) (Conte et al., 2017)		Prior ET (*n* = 344) (Conte et al., 2017)		No prior ET (*n* = 320) (Conte et al., 2017)	
	Ribo + L (*n* = 184)	Pbo + L (*n* = 186)^b^	Ribo + L (*n* = 150)	Pbo + L (*n* = 144)^b^	Ribo + L (*n* = 197)	Pbo + L (*n* = 196)	Ribo + L (n = 69)	Pbo + L (n = 77)	Ribo + L (*n* = 114)	Pbo + L (*n* = 112)^c^	Ribo + L (*n* = 146)	Pbo + L (*n* = 143)	Ribo + L (*n* = 188)	Pbo + L (*n* = 187)	Ribo + L (*n* = 175)	Pbo + L (*n* = 169)	Ribo + L (*n* = 159)	Pbo + L (*n* = 161)
Neutropenia^d^	137 (75)	10 (5)	111 (74)	7 (5)	156 (79.2)	10 (5.1)	44 (63.8)	4 (5.2)	80 (70)	5 (4)	115 (79)	8 (6)	133 (71)	9 (5)	137 (78)	11 (7)	111 (70)	6 (4)
Nausea	92 (50)	52 (28)	80 (53)	42 (29)	111 (56.3)	54 (27.6)	32 (46.4)	23 (29.9)	55 (48)	29 (26)	79 (54)	42 (29)	93 (50)	52 (28)	97 (55)	41 (24)	75 (47)	53 (33)
Fatigue	67 (36)	64 (34)	55 (37)	35 (24)	71 (36.0)	62 (31.6)	27 (39.1)	21 (27.3)	48 (42)	30 (27)	43 (30)	48 (34)	79 (42)	51 (27)	56 (32)	48 (28)	66 (42)	51 (32)
Leukopenia^e^	64 (35)	8 (4)	46 (31)	5 (4)	70 (35.5)	9 (4.6)	21 (30.4)	2 (2.6)	36 (32)	0	53 (36)	9 (6)	57 (30)	4 (2)	65 (37)	11 (7)	–	–
Alopecia	62 (34)	26 (14)	49 (33)	25 (17)	62 (31.5)	31 (15.8)	31 (44.9)	7 (9.1)	45 (39)	17 (15)	46 (32)	24 (17)	65 (35)	27 (14)	52 (30)	25 (15)	59 (37)	26 (16)
Diarrhea	56 (30)	36 (19)	61 (41)	37 (26)	66 (33.5)	40 (20.4)	28 (40.6)	22 (28.6)	32 (28)	24 (21)	48 (33)	27 (19)	69 (37)	46 (25)	70 (40)	31 (18)	47 (30)	42 (26)
Arthralgia	54 (29)	55 (30)	37 (25)	40 (28)	–	–	17 (24.6)	24 (31.2)	25 (22)	37 (33)	–	–	50 (27)	61 (33)	–	–	41 (26)	55 (34)
Vomiting	45 (29)	24 (13)	53 (35)	27 (19)	–	–	–	–	29 (25)	17 (15)	49 (34)	24 (17)	–	–	58 (33)	24 (14)	–	–

*AE* adverse event, *CT* chemotherapy, *ET* endocrine therapy, *L* letrozole, *Pbo* placebo, *Ribo* ribociclib

^a^Additional AEs (≥ 15% in either group) in patients ≥ 65 years of age (ribociclib group vs placebo group): anemia (26% vs 6%), constipation (25% vs 16%), decreased appetite (23% vs 17%), cough (19% vs 19%), peripheral edema (19% vs 12%), hypertension (19% vs 19%), rash (19% vs 8%), urinary tract infection (19% vs 10%), headache (18% vs 15%), liver enzyme elevation (17% vs 6%), asthenia (17% vs15%), back pain (15% vs 21%), and hot flush (15% vs 19%)

^b^Four patients in the Pbo + L group did not receive study treatment

^c^One patient in the Pbo + L arm was randomized but did not receive study treatment

^d^Neutropenia also includes “neutrophil count decreased” and “granulocytopenia”

^e^Leukopenia also includes “white blood cell count decreased”

#### Elderly patients

It is estimated that more than 40% of patients with breast cancer are aged ≥ 65 years [13, 14]. Compared with younger women, breast cancer in elderly women (≥ 65 years old) has been associated with a less aggressive disease course, higher incidence of comorbidities, higher avoidance of surgery, and lower trial enrollment due to exclusion criteria or treatment toxicity [15, 16]. These factors, in addition to age-related functional capability and quality of life, influence treatment decisions.

**Fig. 2:**
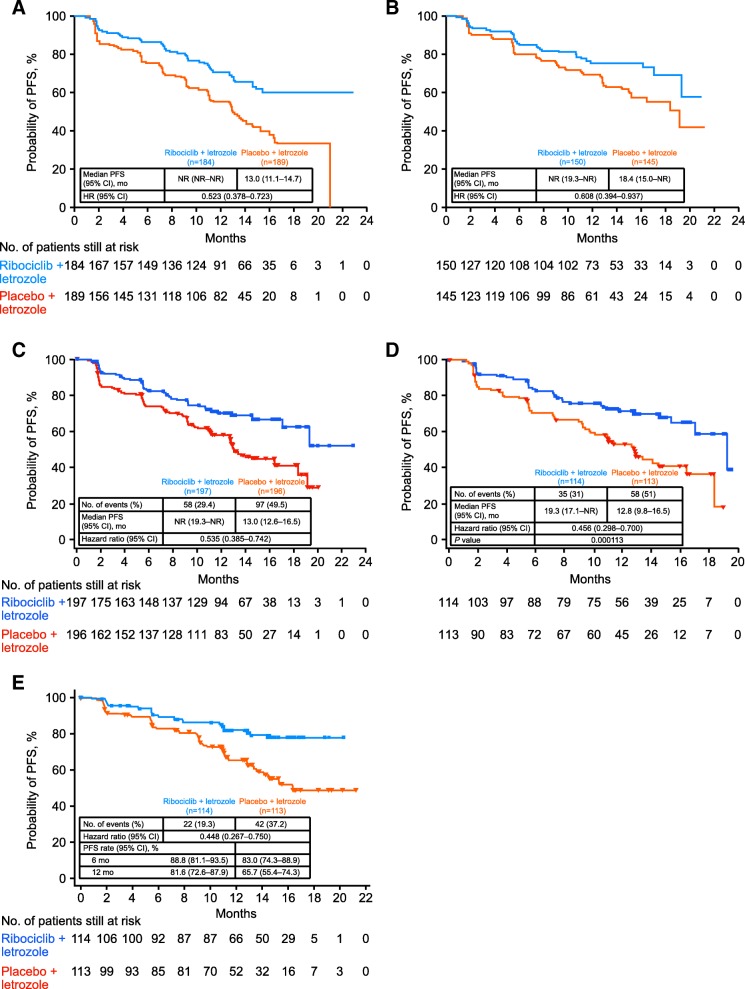
Kaplan-Meier curves showing PFS results for **a** patients aged < 65 years, **b** patients aged ≥ 65 years, **c** patients with visceral metastases, **d** patients with high disease burden, and **e** patients with de novo disease. CI confidence interval, HR hazard ratio, NR not reached, PFS progression-free survival

In MONALEESA-2, 295 patients (44%) were ≥ 65 years of age, of which 150 patients were randomized to receive ribociclib + letrozole; the remaining patients received placebo + letrozole. In patients < 65 years of age, 184 were randomized to the ribociclib group and 186 were randomized to the placebo group. Overall, the baseline characteristics were balanced between patients ≥ 65 and < 65 years of age, except for a higher proportion of ECOG performance status scores of 1 among elderly patients. The combination of ribociclib + letrozole significantly improved PFS compared with placebo + letrozole both in patients ≥ 65 years old (HR = 0.608, 95% CI 0.394–0.937) and in patients < 65 years old (HR = 0.523, 95% CI 0.378–0.723; Fig. 2a, 1b) [17]. In patients ≥ 65 years of age, median PFS was 18.4 months in the placebo + letrozole group vs 13.0 months in patients < 65 years old. Median PFS was not reached in the subsets of patients aged ≥ 65 years and < 65 years in the ribociclib + letrozole group. In patients ≥ 65 years of age, the overall response rate (ORR) in the ribociclib group vs placebo group was 37% vs 31%, compared with 44% vs 25% in patients < 65 years of age.

The safety profile of ribociclib + letrozole in patients ≥ 65 years old was similar to that observed in patients < 65 years old and was consistent with the safety profile of the full population (Table 1) [17]. Grade 3/4 AEs in ≥ 20% of patients in either arm (ribociclib vs placebo) were neutropenia (≥ 65 years, 60% vs 0%; < 65 years, 59% vs 2%) and leukopenia (≥ 65 years, 21% vs 1%; < 65 years, 21% vs 1%); grade 3/4 liver enzyme elevation was reported in 9% vs 2% of patients ≥ 65 years of age and 10% vs 3% of patients < 65 years of age. Treatment discontinuation due to AEs in the ribociclib + letrozole group occurred in 13% and 12% of patients ≥ 65 and < 65 years, respectively. Dose interruptions due to AEs in the ribociclib group occurred in 71% of patients aged ≥ 65 years and in 66% of patients aged < 65 years. Dose reductions due to AEs in the ribociclib + letrozole group occurred in 53% and 49% of patients ≥ 65 and < 65 years, respectively. Neutropenia was the most common AE that led to dose interruptions or reductions in either group. The dose intensity of ribociclib was 86% in patients ≥ 65 years of age and 90% in patients < 65 years of age. In the ribociclib group, 1 patient aged ≥ 65 years experienced grade 3 prolonged QTcF (> 500 ms). Robust PFS data and a low rate of dose reductions and discontinuations suggest that ribociclib + letrozole is an effective first-line treatment option regardless of age.

#### Visceral disease

In MONALEESA-2, 393 patients (59%) had visceral metastases (including liver, lung, and/or other metastatic sites) (Burris HA et al. First-line ribociclib + letrozole in patients with HR^+^/HER2^–^ advanced breast cancer presenting with visceral metastases or bone-only disease: a subgroup analysis of the MONALEESA-2 trial. Poster presented at San Antonio Breast Cancer Symposium, San Antonio, TX, USA; 6–10 December 2016). The primary reason for treatment discontinuation in both patient subgroups was disease progression in 28% vs 47% of patients with visceral disease (ribociclib vs placebo group). Treatment benefit with ribociclib + letrozole was observed in patients with visceral metastases (Burris HA et al. First-line ribociclib + letrozole in patients with HR^+^/HER2^–^ advanced breast cancer presenting with visceral metastases or bone-only disease: a subgroup analysis of the MONALEESA-2 trial. Poster presented at San Antonio Breast Cancer Symposium, San Antonio, TX, USA; 6–10 December 2016) (Fig. 2c). Median PFS was not reached (95% CI 19.3 to not reached) in the ribociclib group and was 13.0 months (95% CI 12.6–16.5) in the placebo group (HR = 0.535; 95% CI 0.385–0.742). In patients with ≥ 3 metastases (high disease burden; Fig. 2d), comparable results were observed (Verma S et al. Ribociclib + letrozole vs placebo + letrozole in postmenopausal women with HR^+^/HER2^–^ advanced breast cancer and a high disease burden. Poster presented at the IMPAKT Breast Cancer Conference, Brussels, Belgium; May 4–6 May 2017). Median PFS was 19.3 months (95% CI 17.1 to not reached) in the ribociclib + letrozole group vs 12.8 months (95% CI 9.8–16.5) in the placebo + letrozole group (HR = 0.456; 95% CI 0.298–0.700). The 12-month PFS rate was 71.5% in the ribociclib + letrozole group vs 53.5% in the placebo + letrozole group. An analysis of best overall response (BOR) per RECIST v1.1 showed that 45% of patients in the ribociclib + letrozole group vs 35% in the placebo + letrozole group had a BOR of complete or partial response.

Ribociclib + letrozole treatment in patients with visceral metastases exhibited a similar safety profile to that observed in the full population, irrespective of disease burden (Burris HA et al. First-line ribociclib + letrozole in patients with HR^+^/HER2^–^ advanced breast cancer presenting with visceral metastases or bone-only disease: a subgroup analysis of the MONALEESA-2 trial. Poster presented at San Antonio Breast Cancer Symposium, San Antonio, TX, USA; 6–10 December 2016; Verma S et al. Ribociclib + letrozole vs placebo + letrozole in postmenopausal women with HR^+^/HER2^–^ advanced breast cancer and a high disease burden. Poster presented at the IMPAKT Breast Cancer Conference, Brussels, Belgium; May 4–6 May 2017) (Table 1). In patients with low disease burden, dose interruptions and reductions for ribociclib (in the ribociclib + letrozole group) were required in 153 (77.7%) and 109 (55.3%) patients, respectively; for placebo (in the placebo + letrozole group), dose interruptions and reductions were required in 79 (40.3%) and 12 (6.1%) patients, respectively (Burris HA et al. First-line ribociclib + letrozole in patients with HR^+^/HER2^–^ advanced breast cancer presenting with visceral metastases or bone-only disease: a subgroup analysis of the MONALEESA-2 trial. Poster presented at San Antonio Breast Cancer Symposium, San Antonio, TX, USA; 6–10 December 2016). Treatment discontinuations were reported in 83 patients (42%) in the ribociclib group, and 111 patients (57%) in the placebo group, of which 8% in the ribociclib group and 2% in the placebo group were related to AEs. The most common AEs leading to discontinuation in the ribociclib group were elevated alanine transaminase (ALT; 4.6%), vomiting (4.1%), elevated aspartate transaminase (AST; 2.5%), and nausea (1.5%). In the high-disease burden subgroup, AEs were the cause of ribociclib dose reductions in 50% of patients (vs 4% with placebo) and were the cause of dose interruptions in 74% and 11% of patients in the ribociclib group and placebo group, respectively (Verma S et al. Ribociclib + letrozole vs placebo + letrozole in postmenopausal women with HR^+^/HER2^–^ advanced breast cancer and a high disease burden. Poster presented at the IMPAKT Breast Cancer Conference, Brussels, Belgium; May 4–6 May 2017). Common AEs in the visceral metastases subset are shown in Table 1. Neutropenia and leukopenia were the most common grade 3/4 AEs irrespective of disease burden. This subanalysis highlights that ribociclib + letrozole can provide significant clinical benefit for patients with visceral metastases.

#### Bone-only disease

Breast cancer may adversely affect the bone health of patients. It is estimated that breast cancer metastasized to the bone in approximately 65% to 85% of patients during the disease course [18, 19]. Bone also represents the first site of metastasis for 26% to 50% of patients with metastatic breast cancer [19], and approximately 70% of patients with advanced or metastatic breast cancer exhibit metastatic bone disease [20]. In the MONALEESA-2 study, results from the bone-only disease subset (ribociclib group, *n* = 69; placebo group, *n* = 78) were similar to those in the overall population (Burris HA et al. First-line ribociclib + letrozole in patients with HR^+^/HER2^–^ advanced breast cancer presenting with visceral metastases or bone-only disease: a subgroup analysis of the MONALEESA-2 trial. Poster presented at San Antonio Breast Cancer Symposium, San Antonio, TX, USA; 6–10 December 2016). The number of PFS events was 18 vs 32 in the ribociclib + letrozole group vs the placebo + letrozole group. The median PFS in patients with bone-only disease was not reached vs 15.3 months in the ribociclib + letrozole group vs placebo + letrozole group, respectively (HR = 0.690; 95% CI 0.381–1.249). A BOR of complete or partial response was observed in 10% of patients in the ribociclib + letrozole group and 4% in the placebo + letrozole group.

Ribociclib + letrozole in patients with bone-only disease had a safety profile consistent with that observed in the full population (Burris HA et al. First-line ribociclib + letrozole in patients with HR^+^/HER2^–^ advanced breast cancer presenting with visceral metastases or bone-only disease: a subgroup analysis of the MONALEESA-2 trial. Poster presented at San Antonio Breast Cancer Symposium, San Antonio, TX, USA; 6–10 December 2016) (Table 1). The most frequent grade 3/4 AEs in the ribociclib + letrozole group (≥ 20% of patients) were neutropenia and leukopenia. Discontinuations due to AEs in the ribociclib + letrozole group were reported in one patient for each of the following AEs: elevated ALT, elevated AST, hepatocellular injury, hepatotoxicity, joint stiffness, depression, and interstitial lung disease. Dose interruptions and reductions were required in the ribociclib + letrozole group in 54 (78.3%) and 35 (50.7%) patients and in the placebo + letrozole group in 30 (39.0%) and 3 (3.9%) patients, respectively. Results from MONALEESA-2 suggest that combination therapy with ribociclib and letrozole may help reduce disease progression in the bone; however, these observations are in a small sample size and need further confirmation in larger subgroups.

#### De novo disease

Patients are classified as having de novo advanced breast cancer if they present with advanced breast cancer but have not been previously diagnosed with an earlier stage of breast cancer, nor have they received prior therapy and relapsed. The benefit of ribociclib + letrozole treatment was maintained in 227 patients (34%) who had de novo advanced breast cancer in the MONALEESA-2 study [21]. Treatment was discontinued in 30% vs 43% of patients with de novo advanced breast cancer in the ribociclib vs placebo groups. In patients with de novo advanced breast cancer, the median relative dose intensity for placebo + letrozole was 100%; the relative dose intensity of ribociclib + letrozole was maintained at 88% despite dose adjustments.

Progression-free survival was prolonged in patients with de novo advanced breast cancer in the ribociclib group vs the placebo group (HR = 0.45; 95% CI 0.27–0.75) (Fig. 2e). Median PFS was not reached in the ribociclib group vs 16.4 months in the placebo group. The 12-month PFS rate in patients with de novo advanced breast cancer was 82% in the ribociclib group vs 66% in the placebo group. In all patients with de novo advanced breast cancer, the ORR (ribociclib vs placebo) was 47% vs 34% and the CBR was 83% vs 77%. Among patients with de novo advanced breast cancer who had measurable disease at baseline, the ORR (ribociclib vs placebo) was 56% vs 45% and the CBR was 82% vs 77% [21].

Ribociclib + letrozole in patients with de novo advanced breast cancer had a similar safety profile to that observed in the full population (Table 1) [21]. The most common grade 3/4 AEs (≥ 20% of patients with de novo advanced breast cancer; ribociclib vs placebo) were neutropenia (55% vs 1%) and leukopenia (21% vs 0); grade 3/4 elevated AST occurred in 6% of patients in the ribociclib group and none in the placebo group. The incidence of elevated ALT events was not reported. Adverse events caused dose reductions in 48% and 5% of patients and caused dose interruptions in 66% and 15% of patients in the ribociclib and placebo treatment groups, respectively. Neutropenia was the most frequent AE leading to dose interruption or reduction (49% of patients in the ribociclib group). Data from MONALEESA-2 suggest that ribociclib provides substantial clinical benefit in the de novo subset, with a safety profile similar to that of the overall population.

#### Prior therapy

It is estimated that approximately 20% to 40% of patients who present with nonmetastatic breast cancer at initial diagnosis will eventually relapse and receive subsequent treatment for recurrent disease [22]. However, the effect of prior (neo)adjuvant treatment on the response to subsequent therapy is unknown [23, 24]. In MONALEESA-2, 220 patients (66%) in the ribociclib + letrozole group and 221 patients (66%) in the placebo + letrozole group had recurrent breast cancer. Overall, a PFS benefit of ribociclib + letrozole vs placebo + letrozole was observed among patients with recurrent breast cancer (HR = 0.60; 95% CI 0.45–0.81) [8]. Furthermore, in an analysis conducted in the updated dataset (data cut-off, 2 January 2017), the PFS benefit of ribociclib treatment was maintained irrespective of the treatment-free interval (TFI) duration (Blackwell KL et al. Subsequent treatment for postmenopausal women with hormone receptor-positive, HER2-negative advanced breast cancer who received ribociclib + letrozole vs placebo + letrozole in the Phase III MONALEESA-2 study. Poster presented at the San Antonio Breast Cancer Symposium, San Antonio, TX, USA; 5–9 December 2017). Ribociclib + letrozole improved PFS vs placebo + letrozole in patients with TFI ≤ 24 months (ribociclib, *n* = 64; placebo, *n* = 72; HR = 0.455; 95% CI 0.296–0.701) and TFI > 24 months (ribociclib, *n* = 85; placebo, *n* = 77; HR = 0.455; 95% CI 0.287–0.720). In patients with TFI ≤ 36 months (ribociclib, *n* = 84; placebo, *n* = 86) and TFI > 36 months (ribociclib, *n* = 65; placebo, *n* = 63), HR (95% CI) was 0.422 (0.284–0.627) and 0.507 (0.303–0.851), respectively. In patients with TFI ≤ 48 months (ribociclib, *n* = 95; placebo, *n* = 100) and TFI > 48 months (ribociclib, *n* = 54; placebo, *n* = 49), HR (95% CI) was 0.449 (0.310–0.650) and 0.496 (0.274–0.898), respectively.

**Fig. 3:**
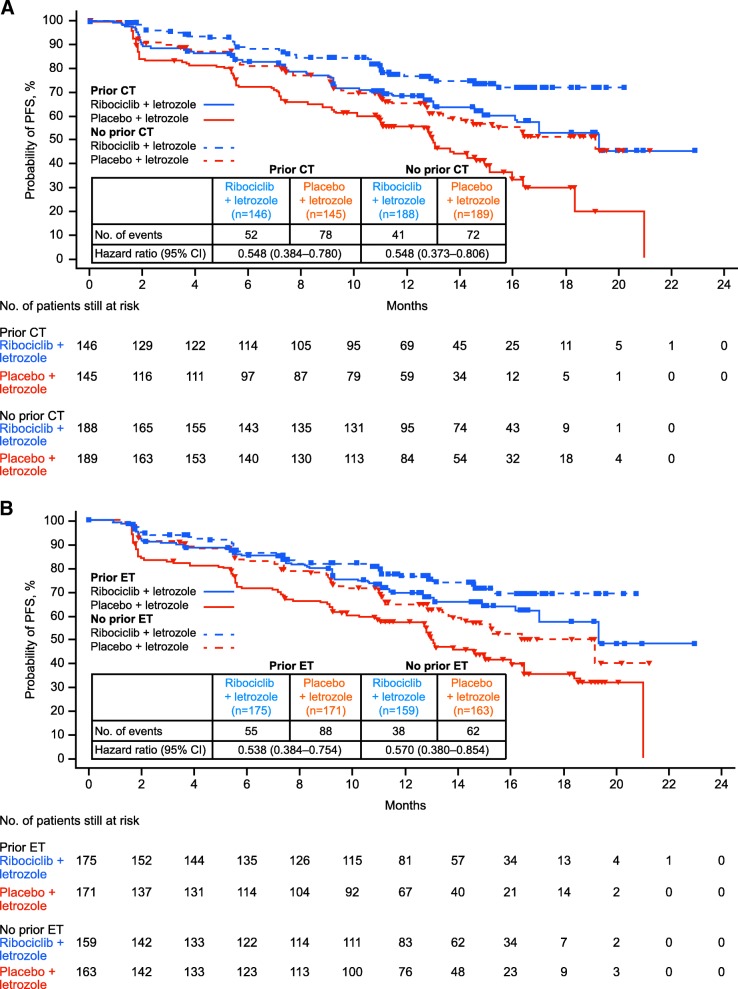
Kaplan-Meier curves showing PFS results for **a** patients with or without prior CT, and **b** patients with or without prior ET in MONALEESA-2. CI confidence interval, CT chemotherapy, ET endocrine therapy, HR hazard ratio, NR not reached, PFS progression-free survival

Progression-free survival was also analyzed according to the type of prior therapy received in the (neo)adjuvant settings (interim PFS cut-off). In the ribociclib group, 146 patients (44%) had prior (neo)adjuvant chemotherapy and 175 patients (52%) had prior (neo)adjuvant ET (Conte P et al. First-line ribociclib + letrozole in patients with HR^+^/HER2^–^ advanced breast cancer who received prior (neo)adjuvant therapy: a subgroup analysis of the MONALEESA-2 trial. Poster presented at the St. Gallen International Breast Cancer Conference, Vienna, Austria; 15–18 March 2017). In the placebo group, 145 (43%) and 171 (51%) patients had prior (neo)adjuvant chemotherapy and (neo)adjuvant ET, respectively. There were 74 patients (37 in each treatment group) who had received a short duration (≤ 14 days) of letrozole or anastrozole for advanced breast cancer prior to enrollment; 36 of these patients had also received prior (neo)adjuvant ET (ribociclib group, *n* = 19; placebo, *n* = 17). Ribociclib significantly increased PFS vs placebo in patients who had received prior (neo)adjuvant chemotherapy (HR = 0.548; 95% CI 0.384–0.780) or ET (HR = 0.538; 95% CI 0.384–0.754) and in patients without prior (neo)adjuvant chemotherapy (HR = 0.548; 95% CI 0.373–0.806) or ET (HR = 0.570; 95% CI 0.380–0.854) (Conte P et al. First-line ribociclib + letrozole in patients with HR^+^/HER2^–^ advanced breast cancer who received prior (neo)adjuvant therapy: a subgroup analysis of the MONALEESA-2 trial. Poster presented at the St. Gallen International Breast Cancer Conference, Vienna, Austria; 15–18 March 2017) (Figs. 3a, b). In patients with prior (neo)adjuvant chemotherapy or ET, median PFS (ribociclib vs placebo) was 19.3 months vs 13.0 months for each of these subgroups. For patients who had not received prior (neo)adjuvant chemotherapy or ET, the median PFS was not reached in the ribociclib group and was 19.2 months for the placebo subgroups. Prior therapy did not appear to influence the response to ribociclib and letrozole based on similarities in HRs of patients with and without previous exposure to the treatment regimen. In patients with prior (neo)adjuvant chemotherapy, the ORR was 38% in the ribociclib group vs 24% in the placebo group; the ORR was 43% and 30% in the ribociclib and placebo group, respectively, in patients with no prior (neo)adjuvant chemotherapy. In patients with prior (neo)adjuvant ET, the ORR was 38% in the ribociclib group and 26% in the placebo group; the ORR was 43% and 29% in the ribociclib and placebo group, respectively, in patients with no prior (neo)adjuvant ET.

The safety profile of ribociclib was consistent with that of other subgroups (Conte P et al. First-line ribociclib + letrozole in patients with HR^+^/HER2^–^ advanced breast cancer who received prior (neo)adjuvant therapy: a subgroup analysis of the MONALEESA-2 trial. Poster presented at the St. Gallen International Breast Cancer Conference, Vienna, Austria; 15–18 March 2017). Dose discontinuations caused by adverse events occurred in 4% and 2% of patients in the ribociclib or placebo group, respectively, who had received prior chemotherapy and 10% and 2% of patients who did not receive prior chemotherapy. Adverse events led to treatment discontinuation in 9% vs 2% of patients with prior ET and 6% vs 3% of patients without prior ET in the ribociclib group vs the placebo group, respectively. Overall, findings from the patient subset with prior therapy suggest that ribociclib is equally effective in patients who received prior therapy for advanced breast cancer and in those who did not. The findings also provide further support for first-line therapy with ribociclib in combination with letrozole for disease recurrence during or after chemotherapy or ET.

#### Subgroup analyses in trials of other CDK4/6 inhibitors and fulvestrant

Direct comparisons of efficacy findings across trials should be generally avoided because of differences in study design that may confound interpretation. Limited data are available from trials of the CDK4/6 inhibitors palbociclib and abemaciclib, as well as fulvestrant, in patients with HR^+^/HER2^−^ advanced breast cancer. Subgroup analyses of PALOMA-1 showed that palbociclib + letrozole also improved median PFS vs letrozole alone across various subgroups such as elderly patients (patients ≥ 65 years), patients with ductal and lobular carcinoma, and patients with metastasis in bone only or in visceral or other sites [25, 26]. However, the overall sample size of the study was much smaller (*n* = 165) than MONALEESA-2, making the inference of any meaningful comparisons challenging. Data regarding efficacy of the different subsets in PALOMA-2 with palbociclib + letrozole treatment are limited and mostly align with the overall efficacy of the trial [7]. Data from subgroup analyses in MONARCH 3 show clinical benefit of abemaciclib + nonsteroidal aromatase inhibitor in most patient subgroups (Goetz MP et al. The benefit of abemaciclib in prognostic subgroups: an exploratory analysis of combined data from the MONARCH 2 and 3 studies. Oral presentation at San Antonio Breast Cancer Symposium, San Antonio, TX, USA; December 5–9 December 2017). Of note, in these exploratory subgroup analyses no PFS benefit with the addition of abemaciclib was found in patients with TFI ≥ 36 months (HR = 0.833; 95% CI 0.457–1.517); in comparison, patients with a TFI > 36 months in MONALEESA-2 did have a PFS benefit with ribociclib + letrozole (Blackwell KL et al. Subsequent treatment for postmenopausal women with hormone receptor-positive, HER2-negative advanced breast cancer who received ribociclib + letrozole vs placebo + letrozole in the Phase III MONALEESA-2 study. Poster presented at the San Antonio Breast Cancer Symposium, San Antonio, TX, USA; 5–9 December 2017). Although efficacy data obtained with most subsets in the FALCON trial were consistent with the overall population and demonstrated superiority of fulvestrant, no PFS benefit of fulvestrant vs anastrozole was observed in patients with visceral metastases (HR = 0.99; 95% CI 0.74–1.33), and the median PFS was shorter in the fulvestrant group (13.8 months vs 15.9 months; *P =* 0.0092) [12]. Thus, future trial outcomes of ribociclib in combination with fulvestrant will be of interest to deduce whether the combination can alter these results.

## Conclusions

The MONALEESA-2 trial demonstrated a clinically meaningful improvement in PFS with ribociclib + letrozole therapy in patients with HR^+^/HER2^−^ advanced breast cancer, and the consistency of the efficacy and safety results was demonstrated across all assessed subgroups. Most of the AEs observed were consistent with the class and were manageable. The incidence of hematologic AEs in the ribociclib + letrozole group was similar across all subgroups. QTc interval prolongation occurred in 3.3% of patients treated at the 600-mg dose of ribociclib, generally within the first 4 weeks of treatment [8]. The study protocol excluded patients at elevated risk for QT interval prolongation; careful monitoring, adequate dose reduction, and dose interruption were implemented as needed [8].

Overall, a retrospective head-to-head comparison of subgroup analyses across trials can be challenging because of differences in trial designs, enrolled patient populations, and unintended patient biases. A key limitation of the MONALEESA-2 trial is the inadequate understanding of the effects of ribociclib over longer periods of time. As the trial is still ongoing, insufficient information currently exists to determine the effect of ribociclib on long-term tolerability and overall survival. Furthermore, based on published literature, there have been fewer clinical trials so far than for palbociclib and, as a consequence, fewer patients have received ribociclib as first-line treatment. However, these shortcomings broadly apply to all the recent advances in first-line therapy of advanced breast cancer.

To address the current limitations and to gain better understanding of the role of ribociclib in different combinations, patient types, and treatment settings, further analysis of the clinical program is required. In the MONALEESA-7 trial (NCT02278120) premenopausal patients were randomized to receive the gonadotropin-releasing hormone agonist goserelin, in combination with a nonsteroidal aromatase inhibitor (letrozole or anastrozole), or tamoxifen, with or without ribociclib, in the first-line setting. The primary endpoint was met, with a median PFS of 23.8 months vs 13.0 months in the ribociclib vs placebo group (HR = 0.553; 95% CI 0.441–0.694; *P* = 9.83 × 10^−8^) (Tripathy D et al. First-line ribociclib or placebo combined with goserelin and tamoxifen or a non-steroidal aromatase inhibitor in premenopausal women with hormone receptor-positive, HER2-negative advanced breast cancer: results from the randomized Phase III MONALEESA-7 trial. Oral presentation at San Antonio Breast Cancer Symposium, San Antonio, TX, USA; 5–9 December 2017). In another ongoing phase 3 trial, MONALEESA-3 (NCT02422615), postmenopausal patients with advanced HR^+^/HER2^−^ breast cancer are randomized to receive fulvestrant with or without ribociclib in the first- or second-line setting. To expand information on the efficacy and safety of ribociclib, a phase 3b open-label, single-arm, multicenter study, CompLEEment-1, will examine efficacy and safety of ribociclib + letrozole in a larger and broader population than MONALEESA-2 (estimated enrollment = 3000 patients).

Overall, the clinically relevant results obtained from the MONALEESA-2 trial suggest that ribociclib in combination with other aromatase inhibitors such as letrozole can be successfully used in the treatment of advanced HR^+^/HER2^−^ breast cancer in a broad population.

## Abbreviations

AE: Adverse event
AKT: Protein kinase B
ALT: Alanine transaminase
AST: Aspartate aminotransferase
BOR: Best overall response
CBR: Clinical benefit rate
CDK: Cyclin-dependent kinase
CI: Confidence interval
ECOG: Eastern Cooperative Oncology Group
ET: Endocrine therapy
FDA: Food and Drug Administration
HER2^–^: Human epidermal growth factor receptor 2-negative
HR^+^: Hormone receptor-positive
HR: Hazard ratio
mTOR: Mammalian target of rapamycin
ORR: Overall response rate
PFS: Progression-free survival
PI3K: Phosphatidylinositol-3-kinase
QTcF: Corrected QT interval (Fridericia’s formula)
TFI: Treatment-free interval
